# Network insights on oxaliplatin anti-cancer mechanisms

**DOI:** 10.1186/2001-1326-1-26

**Published:** 2012-10-29

**Authors:** Osama M Alian, Asfar S Azmi, Ramzi M Mohammad

**Affiliations:** 1 Department of Oncology, Karmanos Cancer Institute, Wayne State University, 4100 John R, HWCRC, Room 732, Detroit, MI, 48201, USA; 2 Department of Pathology, Wayne State University, Detroit, MI, 48201, USA; 3 Hamad, Medical Corporation, Doha, Qatar

**Keywords:** Oxaliplatin, Chemotherapy, Resistance signatures, Systems biology, Network theory, Network modeling

## Abstract

Oxaliplatin has been a crucial component of combination therapies since admission into the clinic causing modest gains in survival across multiple malignancies. However, oxaliplatin functions in a non-targeted manner, posing a difficulty in ascertaining precise efficacy mechanisms. While previously thought to only affect DNA repair mechanisms, Platinum-protein adducts (Pt-Protein) far outnumber Pt-DNA adducts leaving a big part of oxaliplatin function unknown. Through preliminary network modeling of high throughput data, this article critically reviews the efficacy of oxaliplatin as well as proposes a better model for enhanced efficacy based on a network approach. In our study, not only oxaliplatin’s function in interrupting DNA-replication was confirmed, but also its role in initiating or intensifying tumorigenesis pathways was uncovered. From our data we present a novel picture of competing signaling networks that collectively provide a plausible explanation of chemotherapeutic resistance, cancer stem cell survival, as well as invasiveness and metastases. Here we highlight oxaliplatin signaling networks, their significance and the clinical implications of these interactions that verifies the importance of network modeling in rational drug design.

## Review

**Figure 1:**
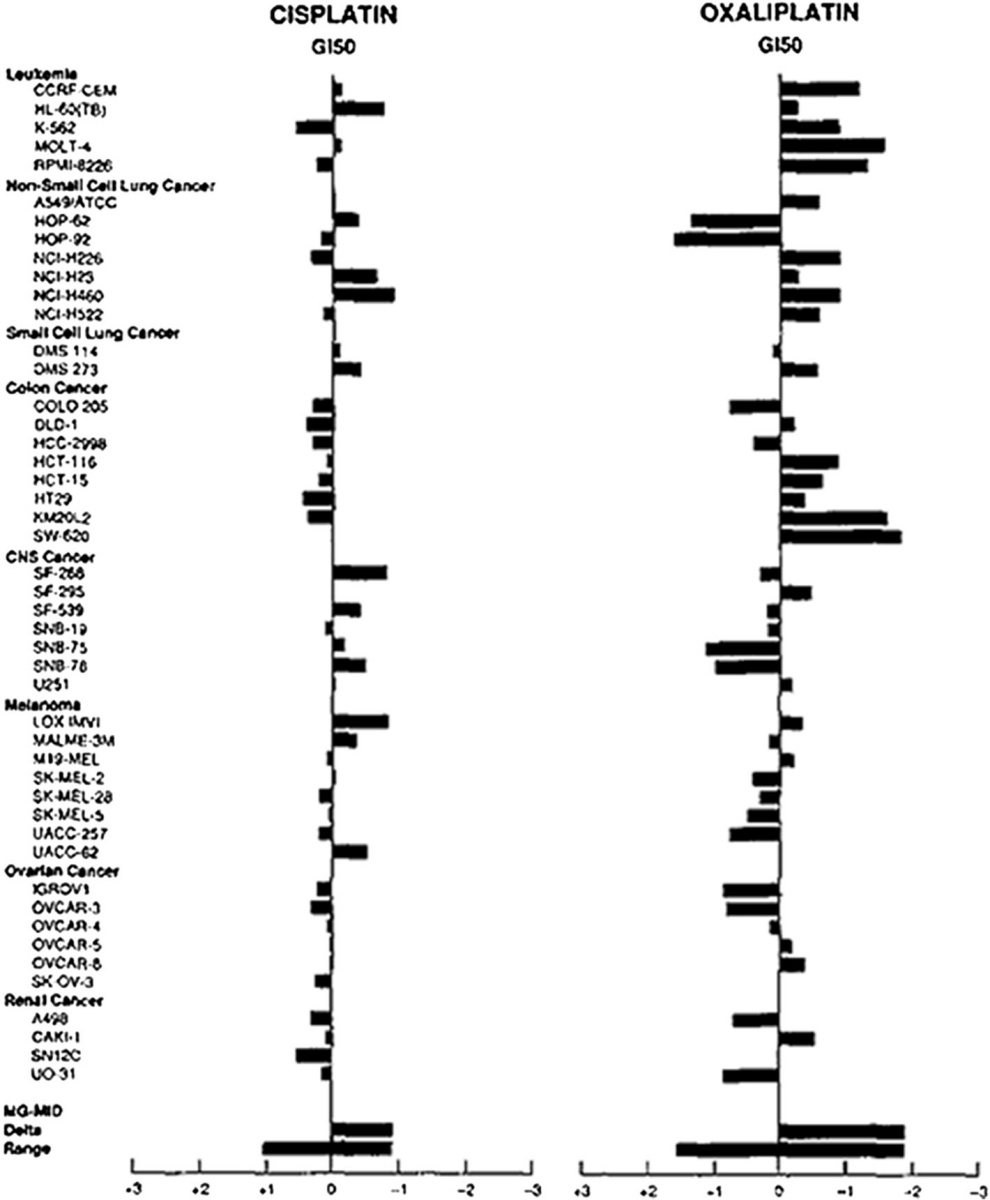
**Oxaliplatin elucidated action mechanism centers around the formation of Pt-GG adducts in DNA.** (**A**). This Pt-DNA NMR structure visualizes the 15 lowest energy solution structures of Oxaliplatin-GG 12-mer DNA adduct (**B**). Figures were adapted from *Protein Interactions with Platinum-DNA Adducts: From Structure to Function*, Chaney et al. 2004 from the Journal of Inorganic Biochemistry (OpenAccess, which allows unrestricted use of the figures).

**Figure 2:**
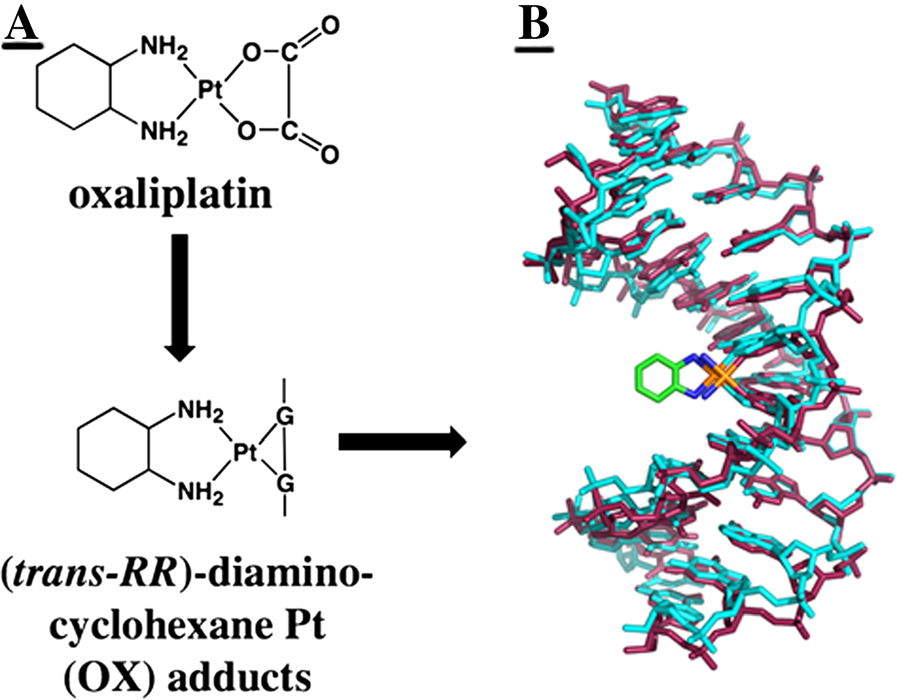
**A comparison of cisplatin and oxaliplatin IG50 results on a standard NCI drug screen panel.** Adapted from *Oxaliplatin, Tetraplatin, Cisplatin, and Carboplatin: Spectrum of Activity in Drug-Resistant Cell Lines and in the Cell Lines of the National Cancer Institute's Anticancer Drug Screen Panel*, Rixe et al. 1996 from the journal Biochemical Pharmacology (OpenAccess, which allows unrestricted use of the figures).

Oxaliplatin, recognized as a DNA intercalating agent, is a platinum coordinated complex that is used in conjunction with different chemotherapies for the treatment of various cancers [1]. In general, oxaliplatin exhibits more efficacious behavior *in vitro* than its close platinum based counterpart, cisplatin. These effects were demonstrated through IG50 experiments on a standard NCI drug screen panel (Figure 1). Oxaliplatin exerts its effects by interfering with DNA replication and transcription machinery through nuclear DNA adduct formation [2]. These Pt-DNA adducts typically are in the form of Pt-Guanine-Guanine (Pt-GG) bonding (Figure 2B) [3]. Ultimately, Pt-DNA complexes at the nucleotide level will either activate DNA repair mechanisms or apoptotic pathways. Interestingly, it has been shown that contrary to its DNA binding capacity, the rate of protein binding of oxaliplatin may be significantly higher than its covalent binding to DNA (in cisplatin adducts, a similarly acting compound, 75-85% of covalent binding occurs with proteins compared with 5-10% in DNA) exhibiting significant differences when compared to DNA lesions [4]. For example, in many cancers there is an over expression of DNA repair proteins such as DNA pol β and knock down or under expression of this protein results in increased sensitivity to oxaliplatin induced DNA damage [5]. These findings build a compelling case for the exploration of protein expression profiles of oxaliplatin treatment, as the specificity of oxaliplatin is not limited to Pt-DNA adducts. Thus, a further examination of oxaliplatin and other platinum compounds used in adjuvant therapies effect on protein expressions is vital to understanding efficacy or lack thereof.

Clinically, the use of oxaliplatin is efficacious only when combined with other agents such as 5-fluorouracil (5-FU) and leucovorin (LV). Prior to entry into US clinics, oxaliplatin was used against colorectal cancer where it did not show, any significant improvement. In relapsed patients treated with both standard 5-FU and LV, oxaliplatin addition to the combination elicited a 9% greater response to treatment than 5-FU/LV infusion alone (0%) and oxaliplatin alone (1% greater response) with an estimated increase of 2 months in median-survival time [6]. Today, this combination, known as FOLFOX, is considered as standard first line treatment of colorectal cancer and trials in other malignancies, such as pancreatic cancer, are continuously being designed. In these combinations, sequence of treatment, treatment duration and cytotoxicity are very dynamic and sensitive resulting in different efficacy rates. Unfortunately, most of these studies involving such combinations have not been able to increase overall survival significantly, further creating a pressing need for new systemic treatments in complex cancer types [7]. Adjuvant therapy using oxaliplatin post-resection in colorectal cancer has modestly increased survival but overall outcome as a single agent in comparison to various treatments remains unknown [8].

Utilizing these findings and in view of the pressing need for newer aggressive treatment models, a preliminary analysis of oxaliplatin treated pancreatic cancer cells was carried out using gene expression microarrays and network modeling. The intention was to determine the primary effects of oxaliplatin on protein interaction networks in these cells. Addressing these protein interactions would help characterize where oxaliplatin succeeds as a single agent or in combination as well as help understand the underlying mechanisms for its failure. In doing so, an elementary model can be formulated describing what pathways and mechanisms have been activated in the cell and through this, develop a predictive model for success or failure of an agent based on already confirmed observations in the knowledge.

### Protein interactions and drug efficacy

Benign cellular pathways are complex, malignant ones more so [9]. One protein’s presence can have a drastic effect on a multitude of proteins and pathways and the interrelationships between seemingly unrelated proteins and pathways is only now coming to light [10]. Interfering with transcription of a single protein or even halting it altogether may have a positive result in patient care, yet over time the disease evolves to activate and upregulate other proteins and pathways rendering some cancers incredibly resistant to chemotherapeutic treatments [11]. What essentially occurs is the network component which has been altered or inhibited by treatment is compensated for by other members of a broader network. Thus, an emerging goal in cancer treatment is not just the targeting of single proteins, but engaging an entire network and its related networks to halt or reverse the disease [12]. Moreover, oxaliplatin has been implicated in Pt-protein adducts, and more and more significance is being placed upon this interaction as something more than just drug inactivation [13]. The effects of oxaliplatin protein adducts remain poorly understood but further research can yield results shedding light on key interactions relating to efficacy and toxicity.

### Oxaliplatin targets protein networks

Initial analyses of high throughput data might suggest at the outset that a drug works through activation of previously elucidated pathways [14]. To address this, we carried out microarray expression profiling and network modeling to understand the oxaliplatin response signatures using a genetically complex pancreatic cancer cell line model. The design and analysis of data has been provided in our previous publications [15,16]. In this study, various mechanisms suggestive of oxaliplatin and its MDM2 inhibitor combination were investigated using systems science. However, single agent oxaliplatin protein network changes were not investigated. Using Capan-2 (wt-p53) model, we investigated the gene expression that was followed by Ingenuity network analysis (IPA) for pathway interactions. Table 1, shows data of known biochemical interactions of oxaliplatin. Among the 35 molecules included within the nucleotide excision repair pathway, nearly 80% were upregulated indicating DNA damage and its concurrent response. Similarly, in 56 molecules related to the RNA polymerase II complex assembly mechanism, nearly 80% were upregulated and nearly 70% of CHK related proteins involved in cell cycle checkpoint control were upregulated with more than 70% of death receptor signaling components activated as well. To our surprise we observed activation in competing carcinogenesis pathways as well. 379 molecules associated with broad cancer promoting mechanisms were found to be upregulated. In Table 2, disease prioritizing analyses showed up-regulation in 45 molecules associated to renal carcinoma, 47 molecules associated to small cell lung cancer, 39 molecules of non-small cell lung cancer signaling, 116 molecules implicated in colorectal cancer signaling, 53 molecules in prostate cancer signaling, 82 molecules of acute myeloid leukemia signaling, 46 molecules in thyroid cancer, 92 molecules of bladder cancer signaling, 65 molecules of pancreatic adenocarcinoma signaling, and 64 molecules of ovarian cancer signaling. Alternate pathways implicated in tumor growth also exhibited upregulation such as VEGF signaling with 49 molecules.

**Table 1:** Based on expression data, molecules relevant to oxaliplatin’s mode of action were activated, shown here

**Multi-targeted effects of oxaliplatin**	
**Signaling network**	**# Up regulated**
Nucleotide Excision Repair	80%
RNA Pol II Complex Assembly	80%
CHK Related Cell Cycle Control	70%
Death Receptor Signaling	70%

**Table 2:** Malignant networks also are partly activated by oxaliplatin treatment, indicating an inherent competition between efficacy and toxicity which can be characterized based on high throughput analysis

**Overview of some signaling changes upon oxaliplatin treatment**			
**Signaling network**	**Molecules in dataset**	**# Up regulated**	**# Down regulated**
Ovarian Cancer	64	34	30
Glioma Signaling	50	26	24
Prostate Cancer	53	31	22
Non-Small Cell Lung Cancer	39	25	14
Colorectal Cancer Metastasis	116	59	57
Basal Cell Carcinoma	25	13	12
Melanoma	25	14	11
Small Cell Lung Cancer	47	28	19
VEGF	49	25	24
Renal Cell Carcinoma	45	30	15
Sonic Hedgehog	15	7	8
Pancreatic Adenocarcinoma	65	37	28

These results point to a remarkable complexity of drug action, but it also speaks to the multiple layers of competition to efficacy that exist. Network theory helps determine the interrelationships between these various pathways and in doing so, better targeting can be achieved simply by understanding the big molecular picture. Many of these pathways feature molecules which are related to other pathways mentioned, whether directly or indirectly. This crosstalk between components is only now being understood in a clinical context and has spurred the development of network pharmacology as a tool to better elucidate drug targets and better design their drugs. The existence of such complications explains the vast difficulties encountered in clinical trials with novel compounds. The nuanced differences between *in vitro* and *in vivo* models for the development of pharmaceuticals have been long known and have expressed themselves in unanticipated toxicities uncovered in initial trials. By further validating the network components of a disease, its relationship to other networks and a compound’s effect on these networks, toxicity can be better predicted based on a consistent system model which may be malleable to each patient’s own unique molecular disease network. Thus, precise response can be better predicted long before a drug enters the clinical trial stage.

Based on the vast body of science available and results from high throughput data, we are able to compile a preliminary explanation in a molecular network sense of why oxaliplatin as a single agent simply does not work (summarized in Table 3). In response to this, a more in depth analysis of combinations with oxaliplatin and other family member compounds such as cisplatin or their combination partners (e.g. FOLFOX) is required to better assess true efficacy in patients. In doing so, better combinations can be developed using already approved compounds present in the market.

**Table 3:** Four molecules were selected based on their fold changes as well as related interacting networks, placing them in the center of a complicated array of cellular events affecting disease progression

**Major metastasis networks activated by oxaliplatin treatment**	
**Signaling node**	**Fold change**
FOS	+2.097
NOTCH	+1.266
FAF	-2.736
VSNL1	-3.343

### FOS signaling – Overexpressed with oxaliplatin treatment

FOS signaling was shown activated with treatment of oxaliplatin in relation to control. The FOS family of proteins consists of four members forming an AP-1 transcription factor complex, FOS, FOSB, FOSL1 and FOSL2. Early studies indicated marked expression of FOS in many malignancies including a possible link between increased c-fos mRNA expression and relapse of acute childhood lymphoblastic leukemia [17]. Similarly, in some non small cell lung carcinoma patients survivability decreased with overexpression of FOS products [18]. Furthermore, recent studies demonstrate the AP-1 complex being at a significant upstream point, possibly regulating the expression of critical pathway signaling components such as the little understood AKR1B10 marker in carcinogenesis [19]. In breast cancer, studies have implicated FOS as a critical member in the activation of MMP-9 by S1P, resulting in enhanced invasiveness and migration of breast cancer cells [20]. Concurrently, FOS was demonstrated as a possible prognostic marker for clinical decision making with BCL2 in endocrine breast cancer tumors [21].

### NOTCH Signaling – Overexpressed with oxaliplatin treatment

**Figure 3:**
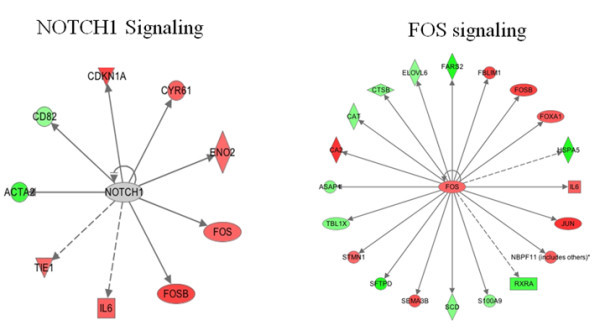
Based on expression data from oxaliplatin treated pancreatic cancer cells, a list of interactions can be surmised and nodes elucidated, prioritizing possible targets for treatment.

Faulty NOTCH expression as well as related family members have been implicated in multiple human diseases since association with T-cell acute lymphoblastic leukemia in 1991 [22]. Many studies have demonstrated higher expression of NOTCH in human malignancies ranging from head and neck cancers, to cervical, lung and pancreatic cancers and also implicating it in epithelial-mesenchymal transition (EMT), a major signal of cancer-stem cells in resistant malignancies [23]. NOTCH signaling has been observed in crosstalk with NF-κB signaling, resulting in increased transcription of NOTCH targets and concurrent increase of carcinogenesis [24]. A multitude of other crosstalk targets have been elucidated, placing NOTCH at the center of a very complicated network. These findings as well as further elucidation of NOTCH signaling downstream effects render it an appealing target in cancer treatment [25]. Figure 3 demonstrates the array of related expression changes of NOTCH signaling with oxaliplatin treatments.

### FAF1 signaling – Under expressed with oxaliplatin treatment

FAF1, a component of the Fas death-inducing signaling complex, was remarkaby under expressed relative to control. Contemporary observations have shown a repeated loss or under expression of FAF1 in some cancers, possibly indicating anti-tumor activity. Experiments have demonstrated a physical interaction between FAF1 and β-catenin whereas an increase in FAF1 resulted in decreased Wnt reporter activity and an increase in β-catenin cytosolic degradation [26]. Simultaneously, FAF1 down regulation has been associated with aberrant NF-κB function.

### VSNL1 signaling – Under expressed with oxaliplatin treatment

A member of the NCS family of proteins, VILIP-1 (a product of the VSNL-1 gene) is found expressed primarily in the tissues of the CNS as well as other peripheral tissues in humans and rats [27]. Various experiments carried out have demonstrated an inverse correlation between VILIP-1 expression and tumor aggression, whereby a down regulation is observed in various human squamous cell carcinomas (SCC) through activation of cAMP- or cGMP-signaling pathways [28]. In one elegant study, transfection of mouse SCC lines with VILIP-1 cDNA resulted in an observable reduction of invasiveness correlating with elevated cAMP levels and reduced MMP-9 and RhoA activity [29]. Loss of VILIP-1 expression paralleled clinicopathological features of SCC in terms of tumor invasiveness as well as local lymph node metastasis. Further evidence has linked VILIP-1 loss with tumor development in neuroblastoma and esophageal SCC and analyses have shown a marked VILIP-1 loss in prostate, lung, ovarian, renal, melanoma and leukemia cancer cells [30]. Taken together, these findings suggest an intricate involvement of VILIP-1 in tumor suppression, of which the precise mechanisms are still being explored.

### Clinical significance and future directions

As drugs are developed and approved, we still lack a coherent and consistent method of predicting efficacy and toxicity in a human model. Based on this preliminary data, we are able to construct roughly a theoretical predictive model of toxicity based on network response. Oxaliplatin serves only as a starting point for determining the veracity of a network approach to drug interactions. Fundamentally, the cancer network is dynamic and drug interactions contribute another complex degree of relationships. While the efficacy of oxaliplatin is not in dispute, the degree of efficacy or efficiency of treatment is something yet unexplored outside this preliminary review.

The degree to which treatment efficiency is successful would be highly dependent on advancements in patient and disease stratification. By characterizing the precise network regulators of a cancer type, further elucidating the nuanced differences between subtypes and further between patients, a targeting motif can be developed. Utilizing this same information, key predictors of patient response can be elucidated, further enhancing clinical efficacy of a drug or guiding combination treatments that may otherwise not already exist. In using oxaliplatin we determined the possibility of incidental activation of undesired networks, competing with the efficacy if the drug. By isolating some of these key nodes, a better and more effective targeting strategy can take place increasing the efficacy of a single agent even with already available treatments used off-label.

## Conclusions

In this study we sought to elucidate the degree of efficacy of oxaliplatin by initially examining high throughput data of oxaliplatin treated capan-2 pancreatic cancer cells. While the efficacy of oxaliplatin was not in debate, the precise cellular mRNA expression profile of single agent treatment had not been previously explored in depth. Preliminary results indicated true to form activation of DNA repair machinery and stress response mechanisms, in line with the established efficacy of oxaliplatin. Surprisingly however, upon treatment several key genes implicated in tumorigenesis, invasiveness and migration displayed either over expression against a desired under expression or vice versa, indicating the activation of pathways competing with oxaliplatin efficacy. Through these preliminary findings, we highlight the importance of oxaliplatin’s effect on FOS, NOTCH, FAF1 and VSNL1 signaling networks representing a sampling of competing pathways. Using network theory and already established protocols and scientific knowledge, efficacy networks can be developed to guide drug development as well as the establishment of combination regimens, increasing the possibility of positive patient response and providing more reliable clinical tools for indicating disease state and treatment efficiency. With the existing technology and knowledge already available, truly personalized treatment is achievable and within reach, possibly spelling the end of cancer lethality within our lifetime.

## Competing interests

The authors declare that they have no competing interests.

## Authors’ contributions

OA carried out data analysis and drafted the manuscript. AA performed the experiments, analyzed the data and edited the manuscript. RM designed the experiments and edited the manuscript. All authors read and approved the final manuscript.
